# Large Low-Field Reversible Magnetocaloric Effect in Itinerant-Electron Hf_1−*x*_Ta*_x_*Fe_2_ Alloys

**DOI:** 10.3390/ma14185233

**Published:** 2021-09-11

**Authors:** Zhao Song, Zongbin Li, Bo Yang, Haile Yan, Claude Esling, Xiang Zhao, Liang Zuo

**Affiliations:** 1 Key Laboratory for Anisotropy and Texture of Materials (Ministry of Education), School of Materials Science and Engineering, Northeastern University, Shenyang 110819, China; 15926343131@163.com (Z.S.); yangb@atm.neu.edu.cn (B.Y.); yanhaile@mail.neu.edu.cn (H.Y.); zhaox@mail.neu.edu.cn (X.Z.); lzuo@mail.neu.edu.cn (L.Z.); 2 Laboratoire d’Étude des Microstructures et de Mécanique des Matériaux (LEM3), CNRS UMR 7239, Université de Lorraine, 57045 Metz, France; claude.esling@univ-lorraine.fr

**Keywords:** magnetocaloric effect, magnetic transition, adiabatic temperature change, laves phase

## Abstract

First-order isostructural magnetoelastic transition with large magnetization difference and controllable thermal hysteresis are highly desirable in the development of high-performance magnetocaloric materials used for energy-efficient and environmental-friendly magnetic refrigeration. Here, we demonstrate large magnetocaloric effect covering the temperature range from 325 K to 245 K in Laves phase Hf_1−*x*_Ta*_x_*Fe_2_ (*x* = 0.13, 0.14, 0.15, 0.16) alloys undergoing the magnetoelastic transition from antiferromagnetic (AFM) state to ferromagnetic (FM) state on decreasing the temperature. It is shown that with the increase of Ta content, the nature of AFM to FM transition is gradually changed from second-order to first-order. Based on the direct measurements, large reversible adiabatic temperature change (Δ*T_ad_*) values of 2.7 K and 3.4 K have been achieved under a low magnetic field change of 1.5 T in the Hf_0.85_Ta_0.15_Fe_2_ and Hf_0.84_Ta_0.16_Fe_2_ alloys with the first-order magnetoelastic transition, respectively. Such remarkable magnetocaloric response is attributed to the rather low thermal hysteresis upon the transition as these two alloys are close to intermediate composition point of second-order transition converting to first-order transition.

## 1. Introduction

Magnetic refrigeration, as an alternative cooling technology with the promises of high-efficiency and environment-friendly, has been recognized as a competitive substitute to replace the conventional gas-compression based refrigeration technology. Magnetic refrigeration is designed on the basis of magnetocaloric effect (MCE) [1], which is an intrinsic magneto-thermodynamic property of magnetic materials, using the isothermal magnetic entropy change (Δ*S_M_*) or the adiabatic temperature change (Δ*T_ad_*) on exposure to a magnetic field as the performance index. From the viewpoint of practical applications, high-performance magnetocaloric materials, especially those with large reversible MCE actuated at relatively low magnetic field (no more than 2 T), are highly sought in accelerating the commercialization process of magnetic refrigeration. In recent years, the utilization of first-order magnetic transition with large magnetization jump to achieve giant MCE has become the focus of discussion. Several representative alloy systems, such as La-Fe-Si [2,3,4], Mn-Fe-P-As (Ge, Si) [5,6,7], Gd-Si-Ge [8,9] and Heusler type Ni-Mn-based alloys [10,11,12,13,14,15], have been well developed. 

In general, the first-order magnetic transition can be categorized into two types [16,17]. One is the magnetostructural transition, where the crystal structure is simultaneously changed in association with the magnetic transition, and the other is the magnetoelastic transition without symmetry breaking. In the case of magnetostructural transition, it is frequently observed that giant magnetocaloric response is achieved for the first application but significantly weakened for the subsequent field cycling [10], suffering from the large thermal hysteresis rendered by the misfits of two lattices. Such irreversibility in MCE thus raises strong reservations in potential applications. In contrast, for the magnetoelastic transition with the isosymmetric characteristic, the shortcomings relevant to the hysteresis and irreversibility can be effectively manipulated through composition tuning [6]. 

An interesting system exhibiting the magnetoelastic transition is the itinerant-electron pseudobinary Hf_1−*x*_Ta*_x_*Fe_2_ alloys with hexagonal (MgZn_2_-type) Laves phase structure, experiencing the iso-structural transition from antiferromagnetic (AFM) state to ferromagnetic (FM) state on decreasing the temperature [18,19,20,21]. In these alloys, the AFM to FM transition temperature is very sensitive to the Ta content, where increasing Ta content results in considerable decrease in the AFM to FM transition temperature [18]. In contrast, the Néel temperature for the transition from paramagnetic (PM) state to AFM state is less influenced by the content of Ta. For the Hf_1−*x*_Ta*_x_*Fe_2_ alloys with *x* = 0.125–0.175, the Néel temperature is located within temperature range from 334 K to 338 K [22]. A detailed phase diagram can be found in the reference [18], where *x* = 0.13 is demonstrated to be close to the critical point for the convergence of AFM, FM and PM states. It is noted that these alloys are known to exhibit the distinct negative thermal expansion behaviors, as the AFM to FM transition is accompanied by the expansion in the lattice [18]. Furthermore, the discontinuity in the magnetization across the AFM to FM transition can also be served as the basis to explore large MCE [20,23,24]. Even though large Δ*S_M_* values have been demonstrated in various alloys [23,24], direct measurements on Δ*T_ad_* are still lack. It is worth mentioning that according to the relation Δ*T_ad_* ≈ −*T*Δ*S_M_*/*C_p_*, a lower specific heat capacity *C_p_* is very favorable to achieve higher Δ*T_ad_* [25]. Thus, when taking into account the relatively low *C_p_* in the Hf_1−*x*_Ta*_x_*Fe_2_ alloys [26], they are very promising candidates to demonstrate considerably large Δ*T_ad_* values. Especially, at the borderline of a first-order and a second-order transition [17], a combination of large Δ*T_ad_* and good cyclic performance can be expected.

In this work, the magnetocaloric properties in the Hf_1−*x*_Ta*_x_*Fe_2_ (*x* = 0.13, 0.14, 0.15, 0.16) alloys were explored. Here, the composition selection was aimed at exploring the MCE in the vicinity of room temperature, based on the phase diagram shown in the literature [18]. Results show that increasing the content of Ta results in the gradual decrease of the AFM to FM transition temperature and also the change of magnetic transition from the second-order to the first-order. For a straightforward evaluation of magnetocaloric properties, the Δ*T_ad_* values were directly measured. In the vicinity of the intermediate composition point of second-order transition converting to first-order transition, large reversible Δ*T_ad_* values of 2.7 K and 3.4 K have been demonstrated under a low magnetic field change of 1.5 T in the Hf_0.85_Ta_0.15_Fe_2_ alloy and Hf_0.84_Ta_0.16_Fe_2_ alloy, respectively, due to the rather low thermal hysteresis upon the first-order magnetic transition.

## 2. Materials and Methods

The polycrystalline alloys with the nominal compositions of Hf_1−*x*_Ta*_x_*Fe_2_ (*x* = 0.13, 0.14, 0.15, 0.16) were prepared by arc-melting under the protection of high purity argon atmosphere, using the high-purity (4N) metal elements as the raw materials. For achieving a good composition homogeneity, each alloy was melted four times. The as-cast alloys were then encapsulated into vacuumed quartz tubes and isothermally annealed at 1273 K for one week, followed by quenching into water. 

The crystal structure analyses were performed by X-ray diffraction (XRD) with Cu-Kα radiation in a Rigaku SmartLab diffractometer (Rigaku, Tokyo, Japan,) equipped with a temperature control attachment. The iso-field (*M*(*T*) curves) and iso-thermal (*M*(*H*) curves) magnetization measurements were carried out in a Quantum Design MPMS-3 system (Quantum Design, San Diego, CA, USA,), using the disc shaped samples with the dimension of *Φ*3 × 1 mm and the weight of ~0.09 g. To reduce the influence of internal demagnetization field, the magnetic field was applied along circular plane. The specific heat capacity (*C_p_*) was measured by the modulated differential scanning calorimetry (DSC) technology (TA-DSC 25). Direct measurements of adiabatic temperature change (Δ*T_ad_*) induced by magnetic field change were performed in a self-designed experimental device. The temperature range for such device is 223–343 K and the magnetic field, produced by NbFeB permanent magnet in Halbach array, is 1.5 T. Since the magnetic field is stationary, the adiabatic magnetization and demagnetization processes are realized through moving the sample into and out of the uniform magnetic field region, where the sample is placed in a movable rod controlled by servo motor. The time for move-in or move-out is 1 s. Thus, the rate of magnetic field change is 1.5 T s^−1^. The temperature change of the sample (dimension: *Φ*10 × 2 mm, ~2 g) induced by magnetic field change applied along circular plane was measured by a thermocouple directly attached to the sample surface. 

## 3. Results

**Figure 1:**
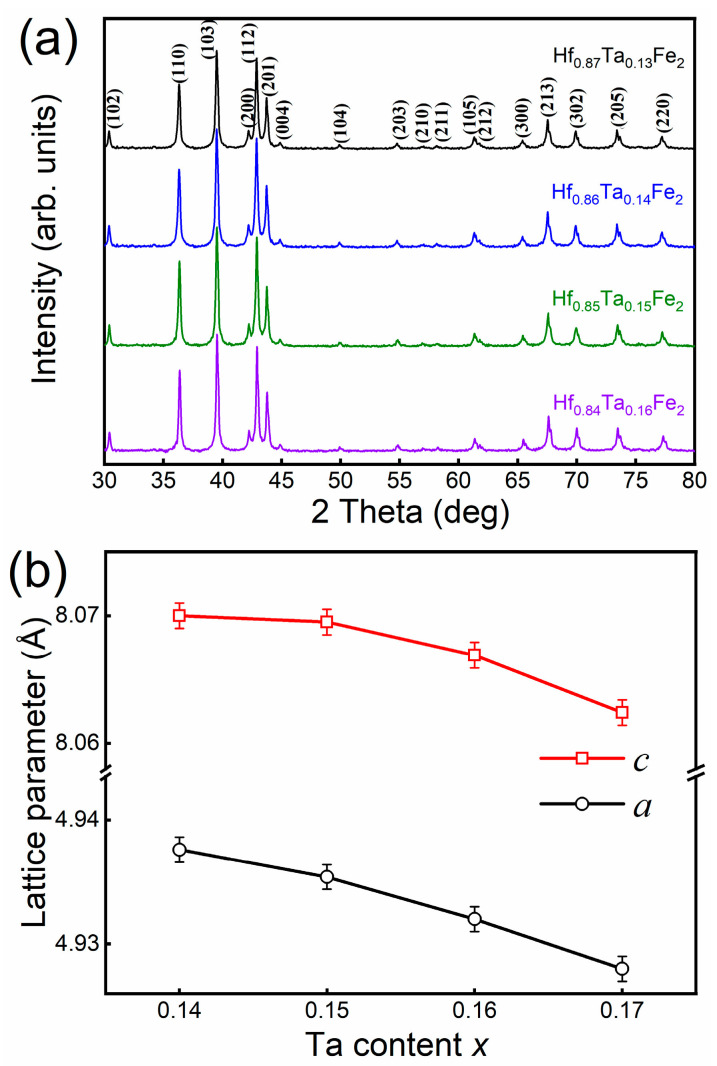
(**a**) Powder X-ray diffraction patterns for the Hf_1−*x*_Ta*_x_*Fe_2_ (*x* = 0.13, 0.14, 0.15, 0.16) alloys; (**b**) Compositional dependence of lattice parameters.

Figure 1a shows the powder XRD patterns for the Hf_1−*x*_Ta*_x_*Fe_2_ (*x* = 0.13, 0.14, 0.15, 0.16) alloys measured at the room temperature. It is seen that all the alloys present the characteristic of hexagonal MgZn_2_-type structure, with the space group of P6_3_/mmc (C14 Laves phase). In the lattice, Fe atoms are expected to be located at 2*a* and 6*h* sites and Hf/Ta atoms at 4*f* site [19]. Figure 1b shows the compositional dependence of lattice parameters for the Hf_1−*x*_Ta*_x_*Fe_2_ alloys as determined from the XRD patterns. With the increase of Ta content, the lattice parameters *a* and *c* almost linearly decrease. The decrease in lattice parameters *a* and *c* indicates the shrink of lattice volume, which should be attributed to the relatively lower atomic radius of Ta (1.43 Å) with respect to that of Hf (1.56 Å).

**Figure 2:**
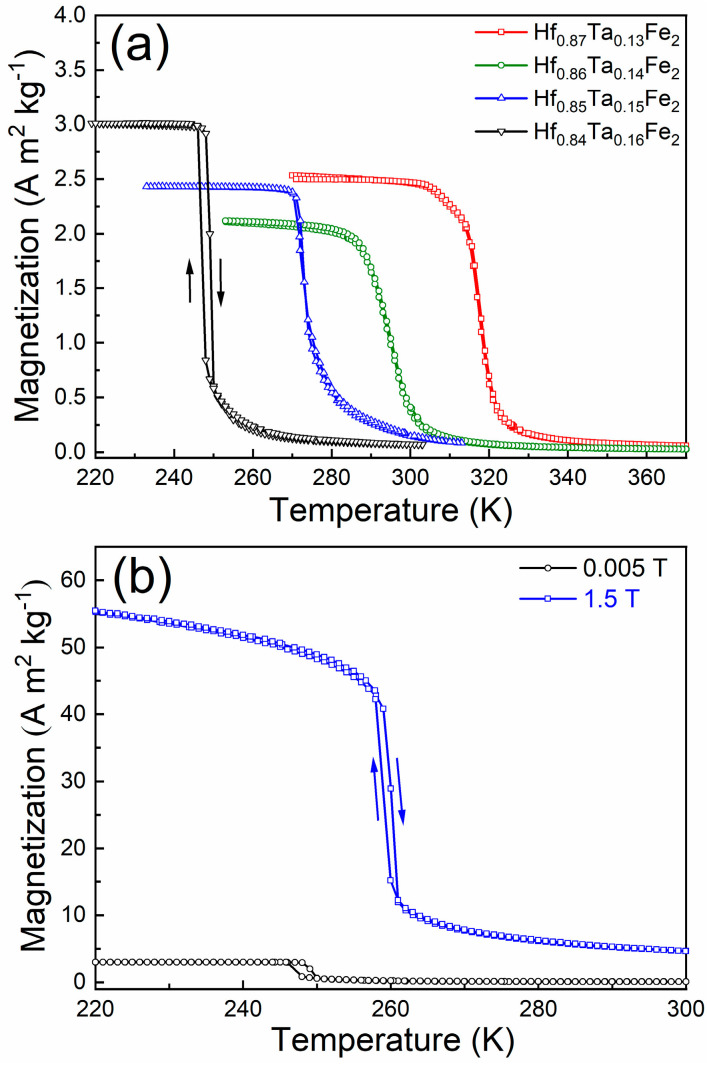
(**a**) Temperature dependence of magnetization (*M*(*T*) curves) under the field of 0.005 T for the Hf_1−*x*_Ta*_x_*Fe_2_ (*x* = 0.13, 0.14, 0.15, 0.16) alloys. (**b**) Comparison on the *M*(*T*) curves under 0.005 T and 1.5 T for the Hf_0.84_Ta_0.16_Fe_2_ alloy.

Figure 2a shows the temperature dependence of magnetization (*M*(*T*) curves) under the field of 0.005 T for the Hf_1−*x*_Ta*_x_*Fe_2_ alloys. For each alloy, the abrupt change in magnetization on cooling corresponds to the transition from high-temperature AFM phase to low-temperature FM phase. Apparently, the AFM to FM transition temperature (*T_t_*) is susceptible to the composition variation and *T_t_* gradually decreases as the increase of Ta content. It is noted that for the Hf_0.87_Ta_0.13_Fe_2_, Hf_0.86_Ta_0.14_Fe_2_ and Hf_0.85_Ta_0.15_Fe_2_ alloys, the *M*(*T*) branch on cooling is almost overlapped with that on heating, indicating the nature of second-order transition. Accordingly, the *T_t_* temperatures for these three alloys are determined to be 323 K (Hf_0.87_Ta_0.13_Fe_2_), 302 K (Hf_0.86_Ta_0.14_Fe_2_) and 277 K (Hf_0.85_Ta_0.15_Fe_2_), respectively. On the other hand, for the Hf_0.84_Ta_0.16_Fe_2_ alloy, a thermal hysteresis of ~2 K between cooling and heating paths can be observed and the averaged *T_t_* is estimated to be 248 K, suggesting the nature of first-order transition. Nevertheless, such thermal hysteresis remains to be quite low, which is conducive to the reversibility of MCE. Based on the *M*(*T*) curves, it is inferred that the increase of Ta content allows a gradual evolution in the nature of magnetic transition from second-order to first-order. 

Figure 2b compares the *M*(*T*) curves under the field of 0.005 T and 1.5 T for the Hf_0.84_Ta_0.16_Fe_2_ alloy. It is evidenced that the AFM to FM transition is accompanied by large magnetization jump. Owing to such magnetization difference, the AFM to FM transition is thus shifted to higher temperature region on increasing the magnetic field, since the magnetic field favors the phase with high magnetization. Under the field of 1.5 T, the AFM to FM transition temperature can be increased by 11 K, with the rate of 7.3 K T^−1^.

**Figure 3:**
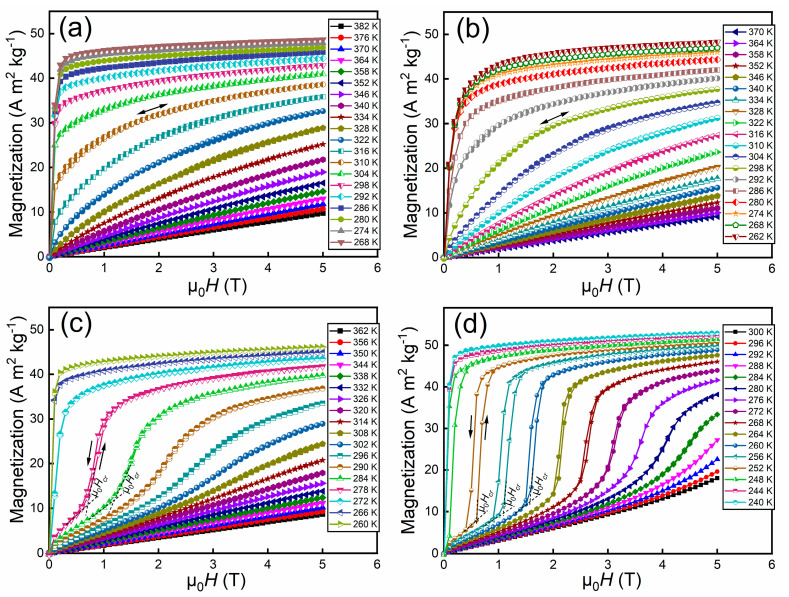
Field-up and field-down isothermal *M*(*H*) curves for the Hf_1−*x*_Ta*_x_*Fe_2_ alloys. (**a**) Hf_0.87_Ta_0.13_Fe_2_; (**b**) Hf_0.86_Ta_0.14_Fe_2_; (**c**) Hf_0.85_Ta_0.15_Fe_2_; (**d**) Hf_0.84_Ta_0.16_Fe_2_.

To acquire further insights into the magnetic transition for the Hf_1−*x*_Ta*_x_*Fe_2_ alloys, field-up and field-down isothermal magnetization (*M*(*H*)) curves across the AFM to FM transition were measured with the maximum field up to 5 T, as shown in Figure 3. The *M*(*H*) curves were measured in a discontinuous protocol. Prior to the measurements at each temperature, the sample was firstly zero field heated to a temperature well above the AFM to FM transition temperature, and then zero field cooled down to the measuring temperature. After that, the field-up and field-down *M*(*H*) curves were measured. For the Hf_0.87_Ta_0.13_Fe_2_ alloy (Figure 3a), typical ferromagnetic behavior can be observed at the temperatures below *T_t_* (i.e., 323 K), where the magnitude of saturation magnetization gradually increases with decreasing the temperature. At the temperatures above 323 K, the field dependence of magnetization tends to exhibit a linear relation, showing the typical characteristic of antiferromagnetic state. It is noted that there is no obvious magnetic hysteresis between field-up and field-down *M*(*H*) curves. The *M*(*H*) curves for the Hf_0.86_Ta_0.14_Fe_2_ alloy exhibit similar characteristics with those of Hf_0.87_Ta_0.13_Fe_2_ alloy, as demonstrated in Figure 3b. 

In the case of Hf_0.85_Ta_0.15_Fe_2_ alloy (Figure 3c), the *M*(*H*) curves exhibit the typical characteristic of ferromagnetism with no obvious magnetic hysteresis at the temperatures below *T_t_* (i.e., 277 K). Above 277 K, step-like magnetization behavior can be observed, where a sudden jump in the slope followed by a rapid increase in magnetization at a certain critical field μ_0_*H_cr_* takes place. This phenomenon is an indication of magnetic field-induced metamagnetic transition from AFM state to FM state. It is noted that magnetic field-induced AFM to FM transition is fully reversible, with very low magnetic hysteresis (e.g., ~0.1 T at 278 K) between the field-up and field-down isothermal magnetization curves. In addition, the critical field μ_0_*H_cr_* to drive the AFM to FM transition is gradually elevated as the increase of temperature. As for the *M*(*H*) curves of Hf_0.84_Ta_0.16_Fe_2_ alloy demonstrated in Figure 3d, sharp step-like magnetization behaviors can be observed above *T_t_*, indicating the occurrence of metamagnetic transition. Even though the magnetic hysteresis is widened when compared to that of Hf_0.85_Ta_0.15_Fe_2_ alloy, it remains in a relatively low level, e.g., ~0.2 T at 252 K.

**Figure 4:**
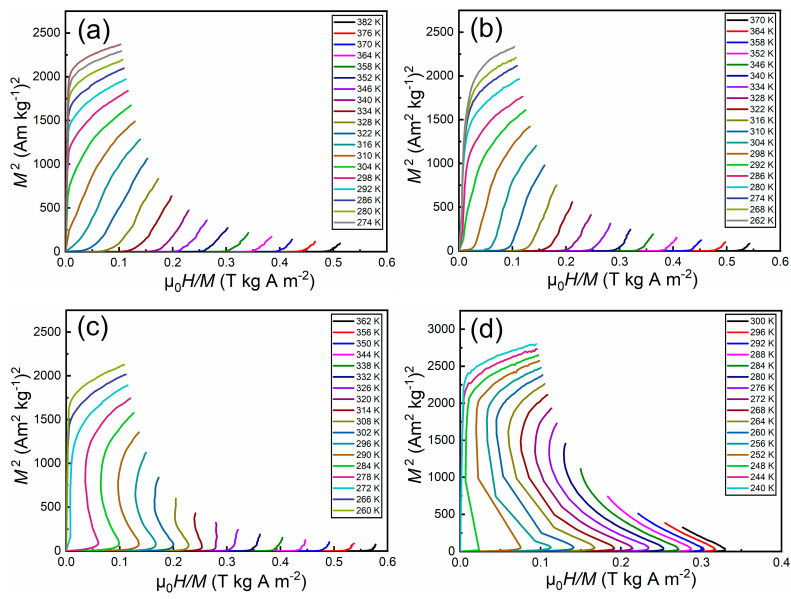
Arrott plots for the Hf_1−*x*_Ta*_x_*Fe_2_ alloys. (**a**) Hf_0.87_Ta_0.13_Fe_2_; (**b**) Hf_0.86_Ta_0.14_Fe_2_; (**c**) Hf_0.85_Ta_0.15_Fe_2_; (**d**) Hf_0.84_Ta_0.16_Fe_2_.

In order to verify the nature of magnetic transition for the Hf_1−*x*_Ta*_x_*Fe_2_ alloys, the Arrott plots were calculated by using the field-up isothermal magnetization curves [27], as shown in Figure 4. Since there is no negative slope for the Arrott plots in Figure 4a,b, the magnetic transition for the Hf_0.87_Ta_0.13_Fe_2_ alloy and the Hf_0.86_Ta_0.14_Fe_2_ alloy can be confirmed to be second-order. In contrast, the typical *S*-shape of Arrott plots manifests the first-order nature of magnetic transition for the Hf_0.85_Ta_0.15_Fe_2_ alloy (Figure 4c) and the Hf_0.84_Ta_0.16_Fe_2_ alloy (Figure 4d). Thus, the change of second-order transition to first-order transition appears at a tricritical point, which should lay somewhere at the composition *x* = 0.14–0.15. 

**Figure 5:**
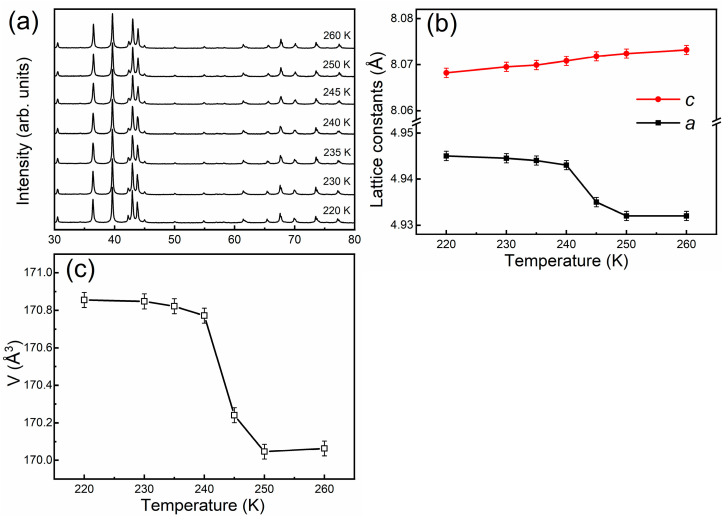
(**a**) Temperature dependent XRD patterns for the Hf_0.84_Ta_0.16_Fe_2_ alloy; (**b**) Change of lattice parameters *a* and *c* as a function of temperature; (**c**) Temperature dependence of unit cell volume.

As the Hf_0.84_Ta_0.16_Fe_2_ alloy exhibits a sharp first-order magnetoelastic transition, temperature dependent XRD measurements were performed in order to acquire deep insights into the crystal structure evolution concomitant with the magnetoelastic transition. Figure 5a shows the temperature dependent XRD patterns for the Hf_0.84_Ta_0.16_Fe_2_ alloy across the first-order AFM-FM transition. It is seen that the hexagonal symmetry for the Hf_0.84_Ta_0.16_Fe_2_ alloy remains unchanged upon the magnetic transition, confirming the characteristic of a magnetoelastic transition. Figure 5b plots the change of lattice parameters *a* and *c* as a function of temperature. On decreasing the temperature, the lattice constant *c* exhibits a gradual decrease in the measured temperature region, i.e., positive thermal expansion. In contrast, a sharp increase in the lattice constant *a* can be observed upon the occurrence of AFM-FM transition, with the ratio ∆*a*/*a* of 0.26%, showing the characteristic of negative thermal expansion. It is noted that the obvious discontinuity in the lattice parameter *a* is a reflection of first-order magnetoelastic transition. Figure 5c shows the temperature dependence of unit cell volume. It is evidenced that the magnetoelastic transition is accompanied by the increase in the unit cell volume and the volume change ∆*V*/*V* is estimated to be 0.51%. Such negative thermal expansion should be attributed to the large discontinuity in the lattice parameter *a*. 

**Figure 6:**
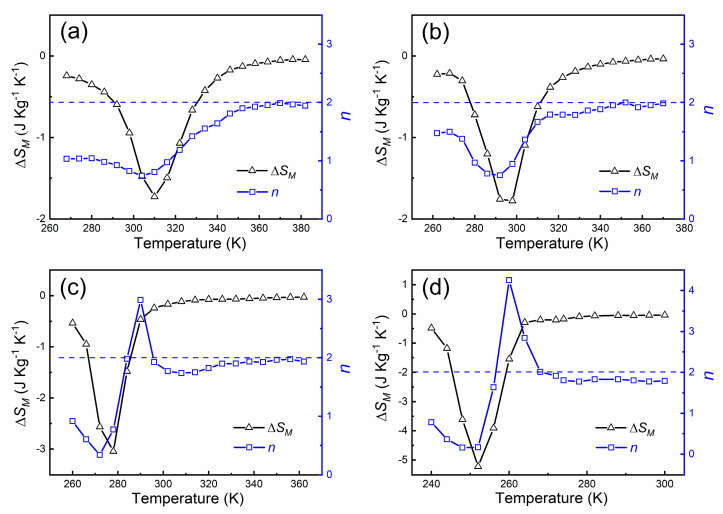
Temperature dependence of Δ*S_M_* and exponent *n* at the field of 1.5 T for the Hf_1−*x*_Ta*_x_*Fe_2_ alloys. (**a**) Hf_0.87_Ta_0.13_Fe_2_; (**b**) Hf_0.86_Ta_0.14_Fe_2_; (**c**) Hf_0.85_Ta_0.15_Fe_2_; (**d**) Hf_0.84_Ta_0.16_Fe_2_.

Based on the field-up *M*(*H*) curves, the magnetic field induced entropy change Δ*S_M_* was calculated by using the Maxwell relation [1]. Under the field change of 1.5 T, the Δ*S_M_* values of −1.72 J kg^−1^ K^−1^, −1.77 J kg^−1^ K^−1^, −3.04 J kg^−1^ K^−1^ and −5.21 J kg^−1^ K^−1^ can be obtained for the Hf_0.87_Ta_0.13_Fe_2_, Hf_0.86_Ta_0.14_Fe_2_, Hf_0.85_Ta_0.15_Fe_2_ and Hf_0.84_Ta_0.16_Fe_2_ alloys, respectively, as demonstrated in Figure 6. In addition, under the field change of 5 T, the corresponding Δ*S_M_* values are −4.0 J kg^−1^ K^−1^ (Hf_0.87_Ta_0.13_Fe_2_), −4.17 J kg^−1^ K^−1^ (Hf_0.86_Ta_0.14_Fe_2_), −4.97 J kg^−1^ K^−1^ (Hf_0.85_Ta_0.15_Fe_2_) and −6.26 J kg^−1^ K^−1^ (Hf_0.84_Ta_0.16_Fe_2_). With the evolution of magnetic transition from second-order to first-order, the maximum Δ*S_M_* values are gradually increased. Moreover, the refrigerant capacity (*RC*) for the present alloys was also determined based on the Δ*S_M_* values [1]. The *RC* values under the field change of 5 T are 135 J kg^−1^, 131 J kg^−1^, 126 J kg^−1^ and 173 J kg^−1^ for the Hf_0.87_Ta_0.13_Fe_2_, Hf_0.86_Ta_0.14_Fe_2_, Hf_0.85_Ta_0.15_Fe_2_ and Hf_0.84_Ta_0.16_Fe_2_ alloys, respectively. 

It has been reported that the order of magnetic transition can also be quantitatively analyzed by calculating the power law exponent *n* based on the Δ*S_M_* values [17], i.e., *n* = dln|Δ*S_M_*|/dln*H*. In the temperature range of magnetic transition, *n* > 2 represents the first-order transition, whereas *n* < 2 indicates the second-order transition. The temperature dependence of the exponent *n* for the present alloys under the field of 1.5 T was also calculated and presented in Figure 6. For the Hf_0.87_Ta_0.13_Fe_2_ and Hf_0.86_Ta_0.14_Fe_2_ alloys, the exponent *n* exhibits a trend of 1→minimum→2, evidencing the second-order transition [17]. For the Hf_0.85_Ta_0.15_Fe_2_ and Hf_0.84_Ta_0.16_Fe_2_ alloys, the magnitude of exponent *n* higher than 2 clearly demonstrates the first-order transition [17]. The determination of magnetic transition order by the exponent *n* is consistent with the results obtained by the Arrott plots.

**Figure 7:**
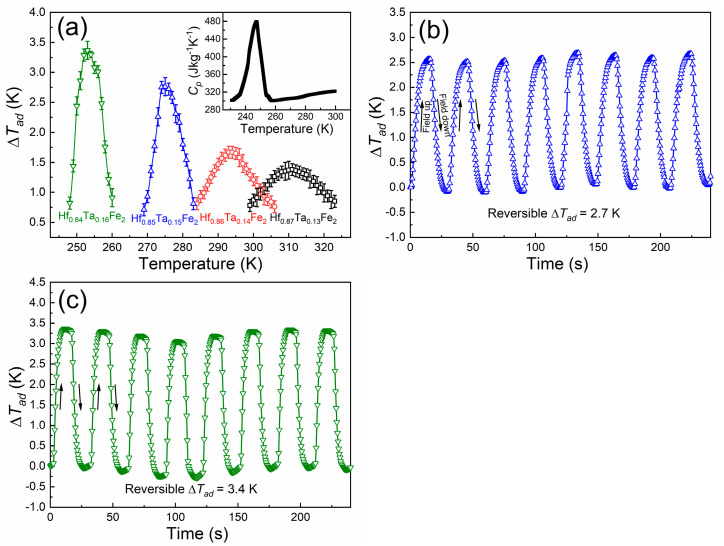
(**a**) Directly measured temperature dependence of Δ*T_ad_* curve for Hf_1−*x*_Ta*_x_*Fe_2_ (*x* = 0.13, 0.14, 0.15, 0.16) alloys under the field change of 1.5 T. The inset shows the specific heat capacity for the Hf_0.84_Ta_0.16_Fe_2_ alloy. The cyclic adiabatic temperature change for (**b**) Hf_0.85_Ta_0.15_Fe_2_ alloy at 275 K and (**c**) Hf_0.84_Ta_0.16_Fe_2_ alloy at 253 K.

Adiabatic temperature change (Δ*T_ad_*), as an important performance index of MCE, allows a straightforward assessment on the magnetocaloric properties [1]. Here, the Δ*T_ad_* values for the studied Hf_1−*x*_Ta*_x_*Fe_2_ alloys under a low field change of 1.5 T were directly measured under the discontinuous protocol. Figure 7a shows the temperature dependence of Δ*T_ad_* values on cooling for the studied Hf_1−*x*_Ta*_x_*Fe_2_ alloys on applying the magnetic field of 1.5 T. For the Hf_0.87_Ta_0.13_Fe_2_ and Hf_0.86_Ta_0.14_Fe_2_ alloys with the second-order magnetic transition, the temperature evolution of Δ*T_ad_* values is moderate and gradual, covering a wide temperature range. Under the field change μ_0_Δ*H* of 1.5 T, the maximum Δ*T_ad_* values of 1.4 K and 1.7 K can be obtained around the AFM-FM transition for the Hf_0.87_Ta_0.13_Fe_2_ and Hf_0.86_Ta_0.14_Fe_2_ alloys, respectively. The temperature dependence of Δ*T_ad_* values for the Hf_0.85_Ta_0.15_Fe_2_ and Hf_0.84_Ta_0.16_Fe_2_ alloys with the first-order magnetic transition is sharp and abrupt, appearing in a relatively narrow temperature range. Accordingly, the maximum Δ*T_ad_* values up to 2.7 K and 3.4 K can be achieved in the Hf_0.85_Ta_0.15_Fe_2_ and Hf_0.84_Ta_0.16_Fe_2_ alloys, respectively. It is shown that the height and width of the Δ*T_ad_* curves for the Hf_1−*x*_Ta*_x_*Fe_2_ alloys are in agreement with the first-order and second-order nature of the magnetic transitions. With the change of magnetic transition from second-order to first-order, the maximum Δ*T_ad_* values are also gradually enhanced, in line with the evolution of Δ*S_M_* values. It should be mentioned that although the Δ*S_M_* values obtained in the present alloys are not very remarkable, the Δ*T_ad_* values are quite impressive, especially for the alloys with the first-order magnetic transition. This effect could be due to the relatively low specific heat capacity *C_p_* for the Hf_1−*x*_Ta*_x_*Fe_2_ alloys (e.g., *C_p_* = ~300 J kg^−1^ K^−1^ for the Hf_0.84_Ta_0.16_Fe_2_ alloy, as shown in inset of Figure 7a), according to the relation Δ*T_ad_* ≈ −*T*Δ*S_M_*/*C_p_*. By using the *C_p_* and the Δ*S_M_* value at 252 K (i.e., −5.21 J kg^−1^ K^−1^) for the Hf_0.84_Ta_0.16_Fe_2_ alloy, the maximum Δ*T_ad_* value can be estimated to be 4.4 K under a magnetic field change μ_0_Δ*H* of 1.5 T, being relatively higher than that obtained by direct measurements.

The reversibility of Δ*T_ad_* for the magnetocaloric materials is of great importance for the potential applications. Considering the characteristic of zero hysteresis for the second-order magnetic transition, Δ*T_ad_* values for the Hf_0.87_Ta_0.13_Fe_2_ and Hf_0.86_Ta_0.14_Fe_2_ alloys are fully reversible. For the Hf_0.85_Ta_0.15_Fe_2_ and Hf_0.84_Ta_0.16_Fe_2_ alloys with the first-order magnetic transition, they also present very good reversibility in the Δ*T_ad_* values during the cyclic magnetization/demagnetization measurements. Figure 7b,c show the reversible behavior of Δ*T_ad_* values for the Hf_0.85_Ta_0.15_Fe_2_ at 275 K and the Hf_0.84_Ta_0.16_Fe_2_ alloys at 253 K under the field change of 1.5 T, respectively. The Hf_1−*x*_Ta*_x_*Fe_2_ alloys exhibit conventional MCE due to the transition from AFM state to FM state on cooling. Thus, the sample warms on magnetization and cools on demagnetization. Stable reversible Δ*T_ad_* value of 2.7 K for the Hf_0.85_Ta_0.15_Fe_2_ alloy and 3.4 K for the Hf_0.84_Ta_0.16_Fe_2_ alloy can be achieved during the cyclic magnetization/demagnetization measurements. Such good reversibility should be attributed to that the rather low thermal hysteresis upon the magnetic transition, since these two alloys lay at the borderline of the first-order transition and the second-order transition. Table 1 compares the present reversible Δ*T_ad_* values with those for some typical magnetocaloric materials. The present reversible Δ*T_ad_* values are superior to those obtained in some alloys with magnetostructural transition. 

## 4. Conclusions

In summary, the magnetoelastic transition and the related magnetocaloric effect in Laves phase Hf_1−*x*_Ta*_x_*Fe_2_ (*x* = 0.13, 0.14, 0.15, 0.16) alloys were investigated. It is shown that the increase of Ta content enables the gradual decrease of the AFM to FM transition temperature and also the conversion from the second-order transition to the first-order transition. Owing to the magnetization difference associated with such AFM to FM transition, the MCE is observed in these compounds in the temperature range from 325 K to 245 K. Under a low magnetic field change of 1.5 T, large Δ*S_M_* values of −3.04 J kg^−1^ K^−1^ and −5.21 J kg^−1^ K^−1^ are obtained in the Hf_0.85_Ta_0.15_Fe_2_ and Hf_0.84_Ta_0.16_Fe_2_ alloys with the first-order magnetic transition. Moreover, large reversible Δ*T_ad_* values up 2.7 K and 3.4 K are also achieved under a low magnetic field change of 1.5 T in the Hf_0.85_Ta_0.15_Fe_2_ and Hf_0.84_Ta_0.16_Fe_2_ alloys, respectively, being much higher than those obtained in some alloys with magnetostructural transition. Such remarkable magnetocaloric properties should be attributed to the rather low thermal hysteresis of first-order magnetic transition in the Hf_0.85_Ta_0.15_Fe_2_ and Hf_0.84_Ta_0.16_Fe_2_ alloys, as they lay at the borderline of the first-order and the second-order magnetic transition. Furthermore, the effect of doping elements will be explored in the following work towards tuning the magnetization difference across the magnetic transition and the resultant magnetocaloric properties.

## Figures and Tables

**Table 1:** Comparisons on the maximum reversible adiabatic temperature changes of some typical magnetocaloric materials reported in the literatures.

Alloys	Transition Type	Temperature (K)	ΔT_cyclic_^max^ (K)	μ_0_Δ*H* (T)	Reference
Hf_0.85_Ta_0.15_Fe_2_	Magnetoelastic	275	2.7	1.5	This work
Hf_0.84_Ta_0.16_Fe_2_	Magnetoelastic	253	3.4	1.5	This work
MnFe_0.95_P_0.595_B_0.075_Si_0.33_	Magnetoelastic	277	2.8	1.1	[6]
Fe_49_Rh_51_	Magnetoelastic	321	6.2	1.9	[28]
Eu_2_In	Magnetoelastic	56	5.0	2.0	[16]
Mn_1.87_Cr_0.13_Sb_0.95_Ga_0.05_	Magnetoelastic	280	1.9	5.0	[29]
Mn_3_GaC	Magnetoelastic	150	3.0	3.0	[30]
MnCo_0.93_Cu_0.07_Ge	Magnetostructural	304	1.1	1.1	[31]
Ni_40_Co_8_Mn_42_Sn_10_	Magnetostructural	298	0.8	1.5	[32]
Ni_45.3_Co_5.1_Mn_36.1_In_13.5_	Magnetostructural	294	1.1	1.5	[33]
Ni_46_Co_3_Mn_35_Cu_2_In_14_	Magnetostructural	272	2.5	1.5	[34]
Ni_50_Mn_35_In_15_	Magnetostructural	286	1.5	1.9	[35]
Ni_45.7_Mn_37.9_Sn_11.8_Co_4.9_	Magnetostructural	330	1.2	1.93	[36]
Ni_45.7_Mn_36.6_In_13.5_Co_4.2_	Magnetostructural	282	3.0	1.95	[37]
Ni_51.3_Mn_32.9_In_15.8_	Magnetostructural	251	0.7	3.0	[38]

## Data Availability

The data presented in this study are available on request from the corresponding author.
